# Estradiol regulates intestinal ABCG2 to promote urate excretion via the PI3K/Akt pathway

**DOI:** 10.1186/s12986-021-00583-y

**Published:** 2021-06-18

**Authors:** Lei Liu, Tianyi Zhao, Lizhen Shan, Ling Cao, Xiaoxia Zhu, Yu Xue

**Affiliations:** 1 Department of Rheumatology, The Second Affiliated Hospital of Zhejiang University, School of Medicine, Zhejiang, People’s Republic of China 310000; 2 Department of Endocrinology, The Second Affiliated Hospital of Zhejiang University, School of Medicine, Zhejiang, People’s Republic of China 310000; 3 Division of Rheumatology, Huashan Hospital, Fudan University, Shanghai, People’s Republic of China 200040

**Keywords:** Estradiol, Intestine, ABCG2, Hyperuricemia, PI3K/Akt

## Abstract

**Objectives:**

The study of sex differences in hyperuricemia can provide not only a theoretical basis for this clinical phenomenon but also new therapeutic targets for urate-lowering therapy. In the current study, we aimed to confirm that estradiol can promote intestinal ATP binding cassette subfamily G member 2 (ABCG2) expression to increase urate excretion through the PI3K/Akt pathway.

**Methods:**

The estradiol levels of hyperuricemia/gout patients and healthy controls were compared, and a hyperuricemia mouse model was used to observe the urate-lowering effect of estradiol and the changes in ABCG2 expression in the kidney and intestine. In vivo and in vitro intestinal urate transport models were established to verify the urate transport function regulated by estradiol. The molecular pathway by which estradiol regulates ABCG2 expression in intestinal cells was explored.

**Results:**

The estradiol level of hyperuricemia/gout patients was significantly lower than that of healthy controls. Administering estradiol benzoate (EB) to both male hyperuricemic mice and female mice after removing the ovaries confirmed the urate-lowering effect of estradiol, and hyperuricemia and estradiol upregulated the expression of intestinal ABCG2. Estradiol has been confirmed to promote urate transport by upregulating ABCG2 expression in intestinal urate excretion models in vivo and in vitro. Estradiol regulates the expression of intestinal ABCG2 through the PI3K/Akt pathway.

**Conclusion:**

Our study revealed that estradiol regulates intestinal ABCG2 through the PI3K/Akt pathway to promote urate excretion, thereby reducing serum urate levels.

**Supplementary Information:**

The online version contains supplementary material available at 10.1186/s12986-021-00583-y.

## Introduction

In humans, urate is the terminal metabolite of purine metabolism. Increased levels (> 420 μmol/L) of serum urate are defined as hyperuricemia [1]. Hyperuricemia may lead to saturation and precipitation of weakly soluble urate as monosodium urate crystals (MSU crystals). MSU crystals deposit in the renal tubules, causing kidney stones, and in the synovial fluid of joints, causing gout, which is the most common form of inflammatory arthritis. Urate in the human body is synthesized mainly by the liver and excreted through the kidneys and intestines [2]. Any abnormality in this process can disturb the urate balance and cause hyperuricemia. Currently, hyperuricemia is thought to be caused mainly by decreased urate excretion. Urate is excreted through the kidneys (70% of the total urate excretion) or the intestines (30%) [3]. The balance between reabsorption and secretion determines the level of urate in the body. Both the secretion and reabsorption of urate rely on transporters. With the development of molecular biology and genetics, various new urate transporters have been discovered, and a deeper understanding of urate dynamics has been gained.

Of the many urate transporters, ATP binding cassette subfamily G member 2 (ABCG2) is of particular interest. It is a high-capacity transporter located mainly at the tip of the brush border of the proximal renal tubules and is essential for the secretion of urate. There is a large amount of genetic evidence suggesting that ABCG2 is clearly related to hyperuricemia. A missense single nucleotide polymorphism (SNP) in *ABCG2* (rs2231142; Q141K) has been shown to be associated with urate concentrations and gout in both white and black individuals [4]. Matsuo et al. [5] compared male gout patients and healthy people in Japan and found two *ABCG2* alleles, Q141K (50% functional residue) and Q126X (without function), with 76.2% of people with gout having ABCG2 dysfunction, and severe ABCG2 dysfunction significantly increased the risk of early-onset gout (RR = 22.2). Among 10 SNPs strongly associated with serum urate concentrations, Zaidi et al. found that the *ABCG2* rs2231142 T allele is strongly associated with early-onset gout [6].

ABCG2 exists mainly in the kidney, intestine and liver. In recent years, the extrarenal urate excretion impact of ABCG2 has received increasing attention. Ichida K et al. [7] found that *ABCG2* gene abnormalities can lead to increased serum urate; however, the amount of urate excreted by the kidneys remained unchanged or even increased. This group also found that the serum urate and creatinine levels of the *Abcg2* knockout mouse model were higher than those of the wild-type control group and that the intestinal urate clearance rate was significantly reduced. Kannangara and colleagues revealed that the *ABCG2* 141 K variant and the fractional renal clearance of urate contribute strongly but independently to hyperuricemia, which provided further evidence of a significant contribution of ABCG2 to extrarenal clearance of urate [8]. A recent study focused on the pathophysiological nature of the common ABCG2 gout and hyperuricemia-associated variant Q141K (rs2231142) and found that participants with the Q141K *ABCG2* variant displayed elevated serum urate, unaltered fractional excretion of uric acid (FEUA), and significant evidence of reduced extrarenal urate excretion. By generating a mouse model of the orthologous Q140K *Abcg2* variant, they also found that male mice had significant hyperuricemia and metabolic alterations but only subtle alterations of renal urate excretion and ABCG2 abundance [9]. The above results all indicate that ABCG2 plays an important role in extrarenal excretion (which mainly occurs via the intestine). Caco-2 cells are commonly used to study intestinal epithelial function. In Caco-2 cells, urate showed a polarized flux from the basolateral to apical side, and this flux was almost abolished in the presence of elacridar, which is an ABCG2 inhibitor, suggesting that the urate transport function of Caco-2 cells depends mainly on ABCG2 [10].

The majority of patients with gout and hyperuricemia are male. In female patients, postmenopausal women have a higher risk of incident gout than premenopausal women [11]. In fact, more than 40 years ago, it was found that estrogen could promote urate excretion and reduce the occurrence of hyperuricemia [12]. Sumino et al. [13] revealed that increased urate levels were closely related to reduced estrogen levels.

ABCG2 was originally discovered in the breast cancer cell line MCF-7/Adr-vp3000, which functions as a transporter to pump chemotherapy drugs out of tumor cells. Breast cancer is a malignant tumor sensitive to sex hormones. Many studies have focused on estrogen-induced breast cancer drug resistance. Most believe that estrogen can increase the expression of ABCG2 in breast cancer cells, thereby increasing drug resistance [14–16]. Since estrogen can regulate the expression of ABCG2 in tumor cells, we wondered whether estrogen can increase the excretion of urate by regulating the expression of ABCG2 and thereby exert an antihyperuricemic effect. However, even though it has been shown that estrogen can regulate the expression of ABCG2 and serum urate, direct administration of estrogen to male gout patients is inappropriate. Therefore, it is important to study the underlying pathway by which estrogen regulates ABCG2 in the excretion of urate.

## Methods

### Patients

In total, eighteen patients with gout (intermittent phase) or hyperuricemia were included in the current study. The patients with gout fulfilled the 1977 American Rheumatism Association (now the ACR) preliminary criteria for acute arthritis of primary gout and the 2015 ACR/European League Against Rheumatism (EULAR) gout classification criteria. The patients who did not fulfill the gout classification and had a serum urate level above 420 μmol/L were defined as having hyperuricemia. The eighteen patients did not start urate-lowering therapy. Seventeen healthy controls were recruited from the physical examination center. Blood samples from the patients were obtained during a clinic visit, and those of the healthy controls were obtained during a physical examination. Serum was separated within 3 h and stored at − 80 °C. The levels of serum urate and subsequent mouse serum urate and urate in vitro were measured with urate assay kits (Sigma-Aldrich, St Louis, MO, USA). Serum estradiol and mouse serum estradiol were measured with an estradiol ELISA kit (Abcam, Cambridge, MA, USA). This study was approved by the Ethics Committee of the Department of Huashan Hospital, Fudan University (No. 2012-137), and the patients provided written consent for their biological material to be used for research.

### Mouse model

C57BL/6 mice were housed at 24 ± 2 °C with 12 h light/12 h dark cycles in a pathogen-free facility at the Central Institute for Experimental Animals of Fudan University. Ad libitum access to food and water was provided. All animal procedures were approved by the ethics committee of the Animal Experiments Committee of Fudan University.

The hyperuricemia mouse model (HUA mice) was established by using yeast polysaccharide (YP) and potassium oxonate (OP) as previously described [17]. The estrogen intervention groups were injected with estradiol benzoate (EB, Selleckchem, Houston, TX, USA, diluted in DMSO), which is more widely used and easily available in clinical practice than estradiol, at 0.2 mg/kg, 1 mg/kg, and 5 mg/kg every other day from the first day of feeding until day 28. There were five mice in each group. During the feeding period, 200 μL of canthal vein blood was collected from each group of mice on days 0, 7, 14, 21 and 28, and the serum was separated within 3 h and stored at − 80 °C. All the mice were sacrificed on day 28. Kidneys and intestines were separated and stored in formalin solution. Subsequently, immunohistochemistry with an anti-ABCG2 antibody was performed on the kidneys and intestines (ileum) of each group of mice. The rabbit anti-human ABCG2 antibody was purchased from Cell Signaling Technology (CST) Company, Danvers, MA, USA. The immunohistochemically stained images were assessed by a semiquantitative four-grade scoring system to evaluate the combination of the staining intensity and the percentage of positive cells. Grade 0 indicated no reaction or a focal weak reaction; grade 1 indicated an intense focal or diffuse weak reaction; grade 2 indicated a moderate diffuse reaction; and grade 3 indicated an intense diffuse reaction.

The ovariectomy (OVX) mouse groups were as follows: the control group with no additional intervention; the OVX group with bilateral ovarian extraction at 4 weeks of age; the sham operation group with the same surgical ovarian exploration as the OVX group at 4 weeks of age, with approximately 1 g of adipose tissue around the ovaries removed but the ovaries left intact; and the OVX + EB group intraperitoneally injected with 8 μg EB every other day after OVX. There were five mice in each group. At 5 weeks of age, the above mouse groups were treated with YP and OP as mentioned above. During the feeding period, 200 μL of canthal vein blood was collected from each group of mice on days 0, 7, 14, 21 and 28, and the serum was separated within 3 h and stored at − 80 °C.

### In vivo intestinal urate excretion model

Three groups of mice were established as follows: the control group with no additional intervention, the HUA mice and the HUA mice treated with 1 mg/kg EB, as mentioned above. There were five mice in each group. The protocol for analyzing intestinal urate excretion was as previously described [7]. On day 21, to analyze intestinal urate excretion, the HUA mice were fasted overnight, anaesthetized by intraperitoneal injection of urethane and cannulated with polyethylene tubing (Hibiki Size 8) (Kunii Co.) at the upper duodenum and the middle jejunum to make an intestinal loop at the upper half of the intestine. After the intestinal contents were removed by the slow infusion of saline and air, efflux buffer (saline containing 0.3 mM potassium oxonate) was introduced into the intestinal loop, and both ends of the loop were closed with syringes. After the indicated periods, the efflux buffer in the loop was collected by syringes, and the urate concentrations were quantified as previously described [7]. Finally, the mice in each group were sacrificed, and the intestinal tissues (ileum, the intestinal tissue with the highest expression of ABCG2 [9]) were collected for western blot analysis.

### In vitro urate transport model

A modified Transwell model was used to analyze urate transport. The filter of the Transwell chamber was a polycarbonate membrane separation medium with a pore size of < 5 µM. After precooling, Matrigel was coated onto the surface of the upper chamber, and Caco-2 cells were cultured for 21 days. The resistance test verified the completeness of the monolayer cell membrane. Two hundred microliters of Hanks solution containing 0.1% BSA was added to the AP side (apical hole) as the intestinal lumen side to reduce the nonspecific adsorption of Caco-2 cells to urate and interventions, and 400 µL of Hanks solution containing urate (with 10^−^ mol/L EB or EB and 5 mmol/L elacridar (Selleckchem, Houston, TX, USA, dissolved in DMSO)) was added to the BL side (basolateral hole) as the base side, after which a resistance test was also carried out. We then placed the 24-well plate with the Transwell chamber in a 37 °C incubator and set different time points to draw 200 µL of the receiving solution from the inner well for testing or freeze-storage at − 20 °C. The operated wells were filled with the corresponding volume of Hanks solution containing 0.1% BSA at 37 °C with a maintained pH of approximately 7.4. The experiment was repeated three times.

### Estradiol and PI3K/Akt regulate ABCG2 expression in Caco-2 cells in vitro

Treatments with 10^–4^, 10^–6^ and 10^–8^ mol/L estradiol and the control (DMSO) were performed, and the Caco-2 cells were exposed for 24, 48 or 72 h. There were 5 wells in each group, and three replicates were carried out. At the end of the intervention, total RNA was extracted from estradiol-treated Caco-2 cells, and Caco-2 mRNA expression was detected by real-time quantitative PCR (qPCR). Then, the Caco-2 cells were treated with 10^–4^ mol/L EB with/without 50 μmol/L LY294002 (Selleckchem, Houston, TX, USA, diluted in DMSO), and the control group was treated with DMSO. The time at which the experimental group showed the most significant increase in mRNA in the above experiments was 48 h. Total RNA was extracted from the Caco-2 cells to detect ABCG2 mRNA expression, and total protein was extracted to detect ABCG2, Akt, p-Akt (S473), and p-Akt (T308) protein expression by western blotting three times. The western blot results were assessed semiquantitatively by ImageJ (v1.52) as previously described [18, 19].

### Real-time quantitative PCR (qPCR) detection system

Total RNA was extracted with TRIzol (Invitrogen, Carlsbad, CA, USA) according to the manufacturer’s instructions, and reverse translation was carried out with an iScript cDNA Synthesis Kit (Bio-Rad, Hercules, CA, USA). The PCR primers (BioTNT, Shanghai, China) used for qPCR were as follows: human *ABCG2*, forward 5′-AGCTGCAAGGAAAGATCCAA-3′ and reverse 5′-TCCAGACACACCACGGATAA-3′, and human *glyceraldehyde-3-phosphate dehydrogenase (GAPDH)*, forward 5′-GGAGCGAGATCCCTCCAAAAT-3′ and reverse 5′-GGCTGTTGTCATACTTCTCATGG-3′. The RNA expression in the samples was normalized to the expression of control housekeeping genes (human *GAPDH*), and the relative mRNA levels of target genes were calculated by the 2^−△△^Ct method.

### Western blot

Cell lysates of different groups were prepared, and equal aliquots of protein extract were electrophoresed by SDS-PAGE. Protein levels were expressed as the ratio of the level of the protein to that of the corresponding GAPDH or β-actin. Rabbit anti-human ABCG2, AKT, phospho-Akt (Thr308), phospho-Akt (Ser473) and β-actin antibodies and rabbit anti-mouse ABCG2 and β-actin, which were used as primary antibodies, were purchased from CST Company.

### Statistical analysis

The data were analyzed with GraphPad Prism 6.01 (GraphPad Software, La Jolla, CA, USA). All experiments were performed at least in triplicate, and the data are expressed as the mean ± standard error of the mean (SEM). Repeated measures analysis of variance (ANOVA) followed by the Student–Newman–Keuls (S–N–K) test were used for post hoc analyses of differences among groups. *P* values < 0.05 were considered to indicate statistically significant differences. The correlation between variables was evaluated by the Pearson correlation test. Two-sided *P* values < 0.05 were considered statistically significant.

## Results

### Serum estradiol and urate levels in patients and healthy controls

**Fig. 1:**
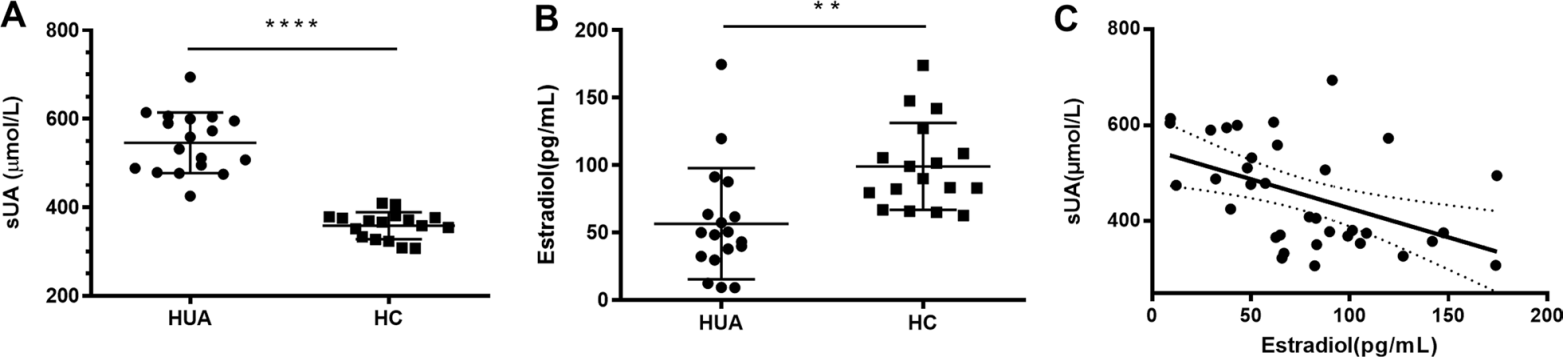
**a** Serum urate levels in patients with gout/hyperuricemia were significantly higher than those in healthy controls (*P* < 0.0001). **b** Hyperuricemia and gout patients had significantly lower serum estradiol levels than healthy controls (*P* < 0.01). **c** Regression analysis of age and levels of serum estradiol and urate. The results showed that age had no significant effect on the levels of serum estradiol and urate (coefficient = 0.024, *P* = 0.886) and that the levels of serum estradiol and urate were significantly negatively correlated (coefficient = − 0.466, *P* = 0.008). *sUA* serum urate, *HUA* hyperuricemia and gout patients

The 18 patients with gout and hyperuricemia in the study were all males of 53.3 ± 15.2 years old, and the healthy controls were males of 49.0 ± 12.3 years old. The serum estradiol level in the hyperuricemia/gout patients was 56.48 ± 41.22 pg/mL, and that in the healthy controls was 98.98 ± 32.24 pg/mL. The serum urate level in the hyperuricemia/gout patients was 545.78 ± 68.39 μmol/L, and that in the healthy controls was 358.35 ± 30.39 μmol/L. The serum urate level in the patients with gout/hyperuricemia was significantly higher than that in the healthy controls (*P* < 0.0001) (Fig. 1a). The gout/hyperuricemia patients had significantly lower serum estradiol levels than the healthy controls (*P* < 0.01) (Fig. 1b). Regression analysis of age and the levels of serum estradiol and urate was performed to correct the effect of age on the relationship between serum estradiol and urate levels. The results showed that age had no significant effect on serum estradiol and urate levels (coefficient = 0.024, *P* = 0.886) and that the levels of serum estradiol and urate were significantly negatively correlated (coefficient = -0.466, *P* = 0.008). The fitting curve of the blood estradiol and urate levels after regression analysis is shown in Fig. 1c, with r = − 0.4743 and *P* = 0.004.

### Male hyperuricemia mouse model with EB intervention

**Fig. 2:**
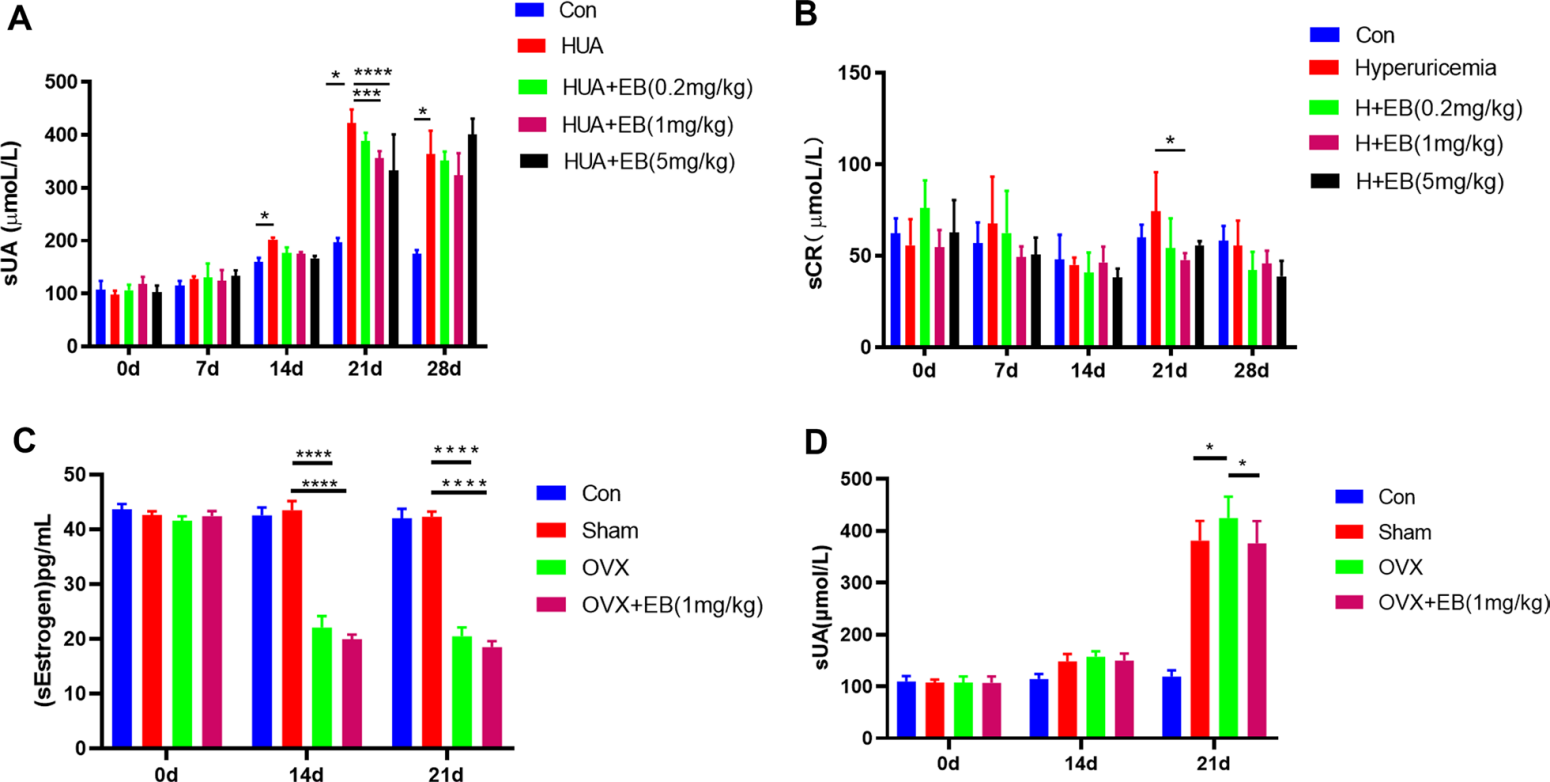
**a** On day 14, the serum uric level was significantly higher than that of the control group (*P* < 0.05). On day 21, the 1 mg/kg (*P* < 0.05) and 5 mg/kg (*P* < 0.001) EB groups exhibited urate-lowering effects. On day 28, there was no obvious urate-lowering effect of EB. **b** The creatinine level of the HUA group was not significantly higher than that of the control group. On day 21, the creatinine level of the mice in the hyperuricemia group was higher (*P* < 0.05). **c** In the OVX group, a significant drop in estradiol levels was observed on day 14 (*P* < 0.0001) and day 21 (*P* < 0.0001). In the OVX + EB intervention group, the level of estradiol also decreased significantly on day 14 (*P* < 0.0001) and day 21 (*P* < 0.0001). **d** A significant increase in the serum urate level in the OVX group (*P* < 0.05) and a urate-lowering effect of EB (OVX + EB vs. OVX, *P* < 0.05) could be observed on day 21

During the establishment of the HUA mice, blood was collected from the canthal vein on days 0, 7, 14, 21, and 28. The serum urate levels are shown in Fig. 2a. From day 14, the serum urate level was significantly higher than that of the control group, indicating that the HUA mouse model was established. On day 21, a urate-lowering effect was observed in the 1 mg/kg and 5 mg/kg EB groups, but there was no significant difference in the urate-lowering effect of these two groups. On day 28, there was no obvious urate-lowering effect of EB.

At 28 days after HUA mouse establishment, the creatinine level of the HUA group was not significantly higher than that of the control group. On day 21, the creatinine level of the HUA mice was higher than that of the control mice, although there was no significant difference, suggesting that hyperuricemia might cause renal damage. Additionally, the higher creatinine level in the EB (1 mg/kg) group was significantly lower than that in the urate group (*P* < 0.05) (Fig. 2b), suggesting that estradiol might have a renal protective effect.

### Female hyperuricemia mouse model with ovariectomy (OVX)

After OVX in female mice, a significant drop in serum estradiol was observed on day 14, and this effect lasted until day 21 (Fig. 2c). In the OVX and EB intervention groups, serum estradiol also decreased significantly, which was related to the fact that the estradiol kit could only detect estradiol but not EB. Although the level of serum estradiol decreased, there was no significant difference in the serum urate level in each group on day 14 of HUA mouse establishment. A significant increase in the level of serum urate in the OVX group and a urate-lowering effect of EB were observed on day 21 (Fig. 2d).

### Expression of ABCG2 in the kidneys and intestines of HUA mice

**Fig. 3:**
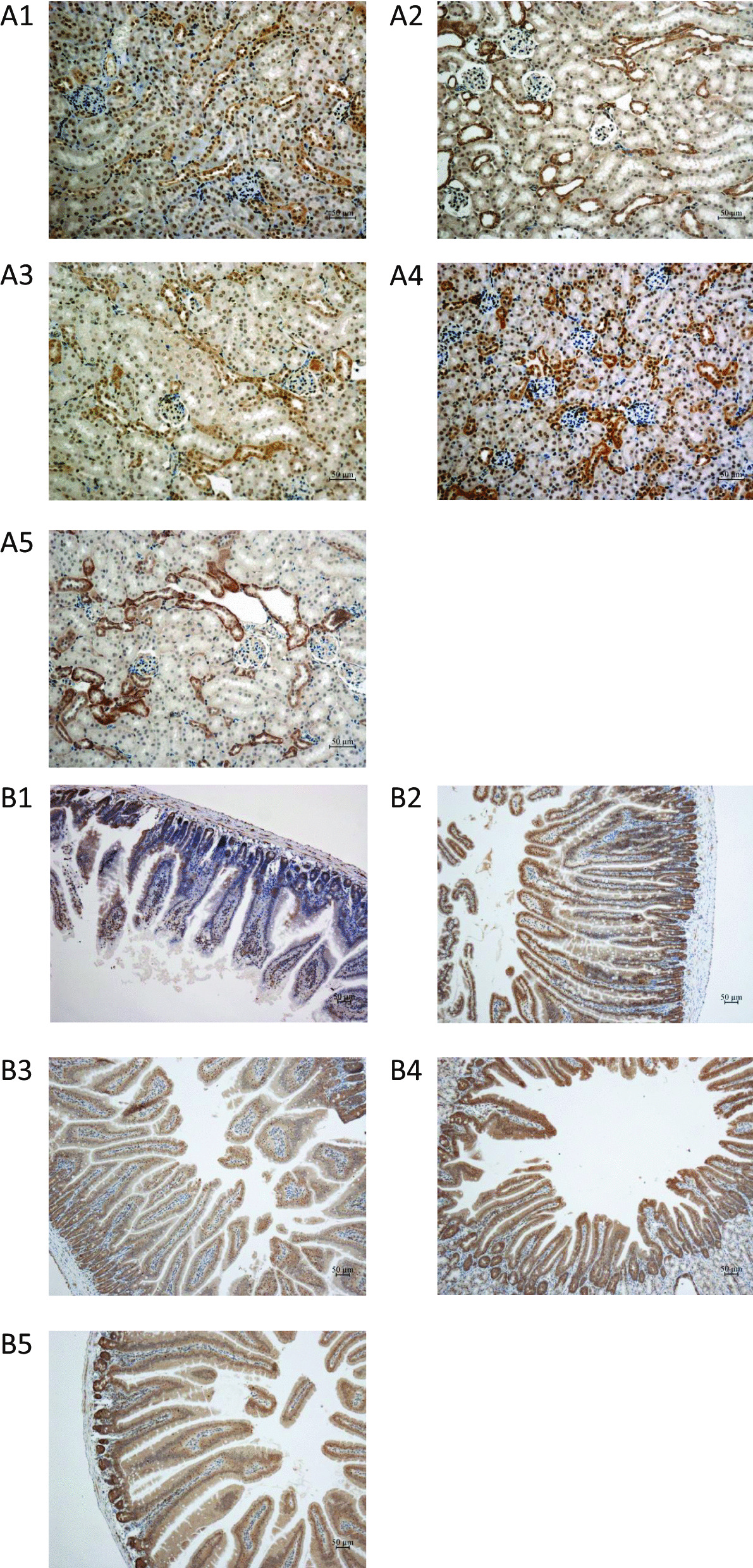
**a1** Renal ABCG2 immunohistochemistry staining of control mice, Grade 1. **a2** Renal ABCG2 immunohistochemistry staining of hyperuricemic mice, Grade 1. **a3**–**a5** Renal ABCG2 immunohistochemistry staining of EB (0.2 mg/kg, 1 mg/kg, and 5 mg/kg) intervention in hyperuricemic mice, Grade 1, Grade 1, Grade 1. **b1** Intestinal ABCG2 immunohistochemistry staining of control mice, Grade 1. **b2** Intestinal ABCG2 immunohistochemistry staining of hyperuricemia mice, Grade 2. **b3–b5** Intestinal ABCG2 immunohistochemistry staining of EB (0.2 mg/kg, 1 mg/kg, and 5 mg/kg) intervention hyperuricemia mice, Grade 3, Grade 3, and Grade 3

Male HUA mice were sacrificed on day 28, and immunohistochemical staining of the kidneys and intestines (ileum) with anti-ABCG2 antibody was performed. There was no obvious difference in the renal expression of ABCG2 between the groups (Fig. 3a1–5). However, intestinal ABCG2 expression in the HUA and EB intervention groups (1 mg/kg and 5 mg/kg) was higher than that in the control group (Fig. 3b1–5).

### Estradiol promotes Caco-2 cell urate transport through ABCG2

**Fig. 4:**
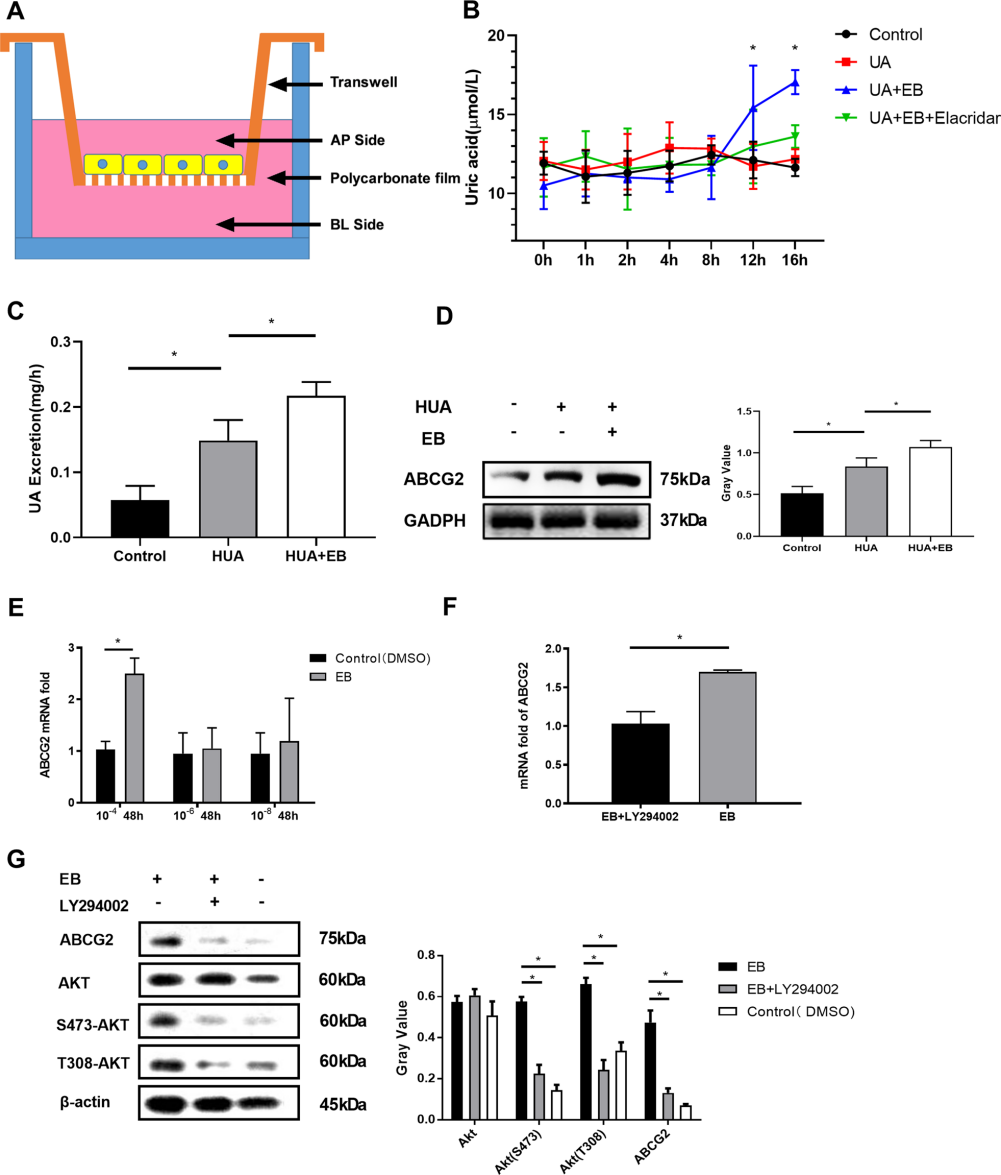
**a** A urate transport evaluation model to evaluate the effect of estradiol on urate transport in Caco-2 cells in vitro. **b** From the 12th hour after the addition of EB, urate transport by Caco-2 cells was significantly increased (*P* < 0.05). When treated with EB and elacridar, which is an ABCG2 inhibitor, the urate transport function of Caco-2 cells was partially weakened (*P* < 0.05). **c** A closed-loop intestinal circulation model to detect the level of intestinal urate excretion. The intestinal urate excretion of the HUA mouse group was significantly increased compared with that of the control mouse group (*P* < 0.05), and the intestinal urate excretion of HUA mice treated with EB was also significantly increased compared with that of the HUA mice (*P* < 0.05). **d** Analysis of ABCG2 protein expression by western blotting in intestinal tissues of each group showed that the intestinal ABCG2 expression of the HUA mice was higher than that in the control mice, and after EB intervention, the intestinal ABCG2 expression of the HUA mice was further increased. **e** Caco-2 cells were treated with different concentrations of EB, and 10^–4^ mol/L EB was found to significantly upregulate ABCG2 mRNA levels without a dose-dependent effect. **f** Caco-2 cells were treated with LY294002 (50 μmol/L) in advance and then treated with EB (10^–^4 mol/L) for 48 h. LY294002 partially blocked the effect of estradiol on the upregulation of ABCG2 mRNA expression (*P* < 0.05). **g** Estradiol did not change the total expression of Akt but regulated ABCG2 by activating p-Akt (S473) and p-Akt (T308), and this activation could also be blocked by LY294002

We established a urate transport evaluation model by simulating the chemotherapy drug tolerance model of tumor cells and accurately evaluated the effect of estradiol on urate transport in Caco-2 cells in vitro (Fig. 4a). From the 12th hour after the addition of EB, urate transport by Caco-2 cells was significantly increased (*P* < 0.05). When treated with EB and elacridar, which is an ABCG2 inhibitor, the urate transport function of Caco-2 cells was partially weakened (*P* < 0.05) (Fig. 4b).

### Estradiol promotes intestinal urate excretion in HUA mice

We constructed closed-loop intestinal circulation in three groups of mice (control group, HUA mice and HUA mice treated with EB), collected 6–7 h of intestinal perfusion fluid, and detected the level of urate in the perfusion fluid. The intestinal urate excretion of the HUA mice was significantly increased compared with that of the control mice (*P* < 0.05), and the intestinal urate excretion of the HUA mice treated with EB was also significantly increased compared with that of the HUA mice (*P* < 0.05) (Fig. 4c). Analysis of ABCG2 protein expression by western blotting in intestinal tissues of each group also showed that the intestinal ABCG2 expression of the HUA mice was higher than that in the control mice, and after EB intervention, the intestinal ABCG2 expression of the HUA mice was further increased (Fig. 4d).

### Estradiol might regulate intestinal ABCG2 expression through PI3K/Akt

We first treated Caco-2 cells with different concentrations of EB and found that 10^–4^ mol/L EB could significantly upregulate the ABCG2 mRNA level of Caco-2 cells at 48 h (the results of different time points are presented in Additional file 1: Fig. 1) without a dose-dependent effect. When we treated Caco-2 cells with LY294002, which is a classic PI3K/Akt inhibitor, in advance and then treated Caco-2 cells with EB, it was found that LY294002 could partially block the effect of estradiol on the upregulation of ABCG2 mRNA expression (*P* < 0.05) (Fig. 4e, f). Akt is partially activated by the phosphorylation of T308, while full activation requires the phosphorylation of S473. To study the underlying mechanism by which estradiol regulates ABCG2 expression, western blotting was used to analyze the expression of ABCG2, Akt, p-Akt (S473) and p-Akt (T308) in each group. Estradiol was also confirmed to up-regulate the expression of ERA and ERB in Caco-2 cells. The results showed that estradiol upregulated the protein expression of ABCG2 in Caco-2 cells, and this effect could be partially blocked by LY294002. Estradiol did not change the total expression of Akt but regulated ABCG2 by activating p-Akt (S473) and p-Akt (T308), and this activation could also be blocked by LY294002 (Fig. 4g).

## Discussion

The prevalence of hyperuricemia in mainland China is approximately 13.3% [20]. Since hyperuricemia is currently a major risk factor threatening people’s health, there is an urgent need to study the metabolism of urate in the human body and explore new targets for urate-lowering therapy. Hyperuricemia is caused mainly by abnormal liver metabolism, urate excretion, or rapid cell turnover. Renal excretion accounts for approximately two-thirds of the total urate excretion, and the remaining one-third is excreted mainly through the intestinal tract. Regulating the secretion and reabsorption of urate in both renal tubules and intestinal tracts are proteins called urate transporters, some of which are currently the therapeutic targets of urate-lowering drugs in clinical use. Among all the transporters, ABCG2 is currently known as the most important transporter for extrarenal urate excretion [7, 9].

Multiple studies have revealed that estradiol-mediated breast cancer resistance is related to alterations in ABCG2 expression [21, 22]. ABCG2 is highly expressed in human placental tissue and might also be regulated by estradiol [23]. Merino et al. [24] found that there were sex differences in the expression and pharmacokinetics of ABCG2 in various organs of mice. The above evidence indicates that the expression and function of ABCG2 are closely related to estradiol. However, there are also some studies that hold different views on the regulation of ABCG2 by gonadal hormones. Tanaka et al. found that ABCG2 expression in mouse liver is stimulated by testosterone [25], and Imai et al.’s research revealed that treatment of MCF-7 cells with estrogen resulted in a drastic decrease in the level of ABCG2 expression [22]. We think that this confirms that gonadal hormones, especially estradiol, have heterogeneity in the regulation of ABCG2 in different tissues, which is one of the original hypotheses of our research.

Research has indicated that there are sex differences in the prevalence of hyperuricemia. Studies by Hak [26] and Sumino [13] have shown that the increase in serum urate levels is closely related to the decrease in estradiol levels in postmenopausal women and that estradiol promotes the excretion of urate. A recent study that first explored the pathophysiological nature of the common *ABCG2* variant Q141K in gout and hyperuricemia revealed that only a male mouse model of the orthologous Q140K *Abcg2* variant had significant hyperuricemia and metabolic alterations with subtle alterations in renal urate excretion and ABCG2 abundance. These mice displayed a severe defect in ABCG2 abundance and function in the intestinal tract [9]. In our study, the first problem investigated was the relationship between serum urate and estradiol in the population. Considering that women’s estrogen levels are related to age and the menstrual cycle, we selected male patients with hyperuricemia and gout and healthy controls as the research subjects and found that the estradiol level of hyperuricemia and gout patients was significantly lower than that of healthy controls. After correcting for age, estradiol and urate levels were significantly negatively correlated. This phenomenon observed in the population has greatly inspired us to continue to explore the underlying mechanism of sex differences in hyperuricemia.

We then verified the urate-lowering effect of estradiol by establishing two different HUA mouse models: treating male mice with EB and removing the ovaries of female mice. The results showed that estradiol could indeed reduce the serum urate of male mice. We also noticed that the effect of estradiol was no longer present after 28 days of treatment. We think that this may be related to the mouse model of hyperuricemia. Since hyperuricemia in mice was most obvious on day 21, the urate-lowering effect of EB was also the most significant. After OVX in the female mice, the level of serum estradiol decreased, and the level of HUA mouse serum urate increased. When EB was given to the OVX mice, the serum urate level decreased, but the level of serum estradiol was not altered since the ELISA kit for estradiol could not detect EB. These two groups of HUA mice fully proved the urate-lowering effect of estradiol. We previously found that Sirt1 can decrease serum urate and upregulate the expression of ABCG2 in the intestine but not the kidneys [27]. Hoque et al. [9] also suggested a tissue-specific pathobiology of the *Q141K* variant, supporting an important role of ABCG2 in urate excretion in the human intestinal tract. More interestingly, Hoque et al. revealed that in female Q140K^+/+^ mice (humanized for *Q141K*), there was no hyperuricemia, while male Q140K^+/+^ mice showed hyperuricemia and impaired renal and intestinal urate excretion, and they believed that this was related to the difference in the abundance of ABCG2 in the kidneys and intestines between male and female mice. Studies of the breast cancer drug resistance mechanism revealed that ABCG2 could pump chemotherapeutic drugs out of tumor cells, and estradiol enhanced this pumping effect. Therefore, we hypothesized that estradiol could also upregulate intestinal ABCG2 to promote intestinal urate excretion.

The immunohistochemical results of HUA mouse kidneys and intestinal tracts suggested that hyperuricemia increased intestinal ABCG2 expression and that estradiol could further upregulate the expression of ABCG2 in the intestine. However, neither hyperuricemia nor estradiol changed the renal expression of ABCG2. After clarifying that estradiol could indeed upregulate ABCG2 expression in the intestine of hyperuricemic mice, we designed two experiments to investigate whether ABCG2 induced by estradiol could promote urate excretion in vitro and in vivo. We first demonstrated that Caco-2 cells expressed estrogen receptors, which could be induced by EB (Additional file 1: Fig. 2). Modified from a model of drug transport in tumor cells, we established a urate transport model of Caco-2 cells in vitro and found that estradiol could promote urate transport in Caco-2 cells, and this effect could be partially blocked by elacridar, as an ABCG2 inhibitor. The results suggested that the upregulation of ABCG2 induced by estradiol could indeed promote urate transport in Caco-2 cells. In the closed-loop model of the intestinal tract, intestinal juice was directly collected and provided a more intuitive observation that estradiol promoted the excretion of intestinal urate by inducing the expression of ABCG2. The above two experiments indicated that estradiol could upregulate the intestinal expression of ABCG2 and confirmed the regulatory effect of estradiol from the perspective of the urate transport function of ABCG2.

The renal and intestinal tissues of HUA mice as well as the intestinal urate transport function models in vivo and in vitro indicated that estradiol could promote intestinal urate excretion by upregulating the intestinal expression of ABCG2. We discovered the regulatory effect of estradiol on the expression and function of intestinal ABCG2; however, the evidence did not completely directly support this conclusion. Since we lacked ABCG2-deficient mice, especially intestinal-specific ABCG2-deficient mice, we could not directly prove the effect of estradiol on intestinal ABCG2 in vivo. We are now trying to establish intestinal-specific ABCG2-deficient mice by the Cre-flox system and aim to collect more convincing data in future research. Most patients with hyperuricemia and gout are male, and if male patients are treated with estradiol, there might be major problems in terms of ethics and patient acceptance. Therefore, the study of estradiol in regulating ABCG2 expression and downstream pathways is essential to understand its effect mechanism and develop new therapeutic targets.

PI3K/Akt is an important pathway that regulates multiple life activities. The main function of Akt relies on mediating different downstream pathways and exerting various actions, such as regulating cell survival, metabolism, and proliferation, by affecting the activation states of various downstream effectors. Studies have found that PI3K/Akt can regulate the expression of ABCG2 in tumor cells [28], and estradiol has a clear role in regulating the PI3K/Akt pathway [29]. Therefore, we studied the specific pathways of estradiol acting on ABCG2 in Caco-2 cells. The results suggest that PI3K/Akt blocked the effect of ABCG2 upregulation by estradiol, which simultaneously phosphorylated two important sites of Akt.

## Conclusion

We have confirmed a urate-lowering effect of estradiol exerted by promoting intestinal urate excretion. The underlying mechanism is that estradiol induces intestinal ABCG2 expression through the PI3k/Akt pathway. Our research provides a theoretical basis for understanding the sex differences in hyperuricemia and gout and new ideas for further research on the therapeutic targets of urate-lowering drugs.

## Supplementary Information

**Additional file 1: Fig. 1**. Different time points of EB treated Caco-2 cells. 10^−4^, 10^−6^ and 10^−8^ mol/L estradiol and control(DMSO) groups were set at different time points 24h (A), 48(B) and 72h(C). ABCG2 mRNA expression was significantly induced by 10^−4^ mol/L EB at 48hrs. **Fig. 2**. Expression of estrogen receptors A and B (ERA and ERB) on Caco-2 cells. Caco-2 cells naturally express ER, and EB could up-regulate the expression of ERA and ERB in Caco-2 cells.

## Data Availability

The datasets supporting the conclusions of this article can be made available upon request.

## Abbreviations

MSU crystals: Monosodium urate crystals
HUA mice: Hyperuricemia mice
YP: Yeast polysaccharide
PO: Potassium oxonate
CST: Cell signaling technology
OVX: Ovariectomy
EB: Estradiol benzoate
HC: Healthy control
ABCG2: ATP binding cassette subfamily G member 2
Ser: Serine
Thr: Threonine

## Code availability

The software application is available.

## References

[1] Major TJ, Dalbeth N, Stahl EA, Merriman TR (2018). An update on the genetics of hyperuricaemia and gout. Nat Rev Rheumatol.

[2] Dalbeth N, Merriman TR, Stamp LK (2016). Gout. Lancet.

[3] Roch-Ramel F, Werner D, Guisan B (1994). Urate transport in brush-border membrane of human kidney. Am J Physiol.

[4] Dehghan A, Kottgen A, Yang Q, Hwang SJ, Kao WL, Rivadeneira F (2008). Association of three genetic loci with uric acid concentration and risk of gout: a genome-wide association study. Lancet.

[5] Matsuo H, Ichida K, Takada T, Nakayama A, Nakashima H, Nakamura T (2013). Common dysfunctional variants in ABCG2 are a major cause of early-onset gout. Sci Rep.

[6] Zaidi F, Narang RK, Phipps-Green A, Gamble GG, Tausche AK, So A (2020). Systematic genetic analysis of early-onset gout: ABCG2 is the only associated locus. Rheumatology (Oxford).

[7] Ichida K, Matsuo H, Takada T, Nakayama A, Murakami K, Shimizu T (2012). Decreased extra-renal urate excretion is a common cause of hyperuricemia. Nat Commun.

[8] Kannangara DR, Phipps-Green AJ, Dalbeth N, Stamp LK, Williams KM, Graham GG (2016). Hyperuricaemia: contributions of urate transporter ABCG2 and the fractional renal clearance of urate. Ann Rheum Dis.

[9] Hoque KM, Dixon EE, Lewis RM, Allan J, Gamble GD, Phipps-Green AJ (2020). The ABCG2 Q141K hyperuricemia and gout associated variant illuminates the physiology of human urate excretion. Nat Commun.

[10] Hosomi A, Nakanishi T, Fujita T, Tamai I (2012). Extra-renal elimination of uric acid via intestinal efflux transporter BCRP/ABCG2. PLoS ONE.

[11] Hak AE, Curhan GC, Grodstein F, Choi HK (2010). Menopause, postmenopausal hormone use and risk of incident gout. Ann Rheum Dis.

[12] Nicholls A, Snaith ML, Scott JT (1973). Effect of oestrogen therapy on plasma and urinary levels of uric acid. Br Med J.

[13] Sumino H, Ichikawa S, Kanda T, Nakamura T, Sakamaki T (1999). Reduction of serum uric acid by hormone replacement therapy in postmenopausal women with hyperuricaemia. Lancet.

[14] Fadok VA, Bratton DL, Konowal A, Freed PW, Westcott JY, Henson PM (1998). Macrophages that have ingested apoptotic cells in vitro inhibit proinflammatory cytokine production through autocrine/paracrine mechanisms involving TGF-beta, PGE2, and PAF. J Clin Invest.

[15] Glavinas H, Kis E, Pal A, Kovacs R, Jani M, Vagi E (2007). ABCG2 (breast cancer resistance protein/mitoxantrone resistance-associated protein) ATPase assay: a useful tool to detect drug-transporter interactions. Drug Metab Dispos.

[16] Takabe K, Kim RH, Allegood JC, Mitra P, Ramachandran S, Nagahashi M (2010). Estradiol induces export of sphingosine 1-phosphate from breast cancer cells via ABCC1 and ABCG2. J Biol Chem.

[17] Chen H, Zheng S, Wang Y, Zhu H, Liu Q, Xue Y (2016). The effect of resveratrol on the recurrent attacks of gouty arthritis. Clin Rheumatol.

[18] Gassmann M, Grenacher B, Rohde B, Vogel J (2009). Quantifying Western blots: pitfalls of densitometry. Electrophoresis.

[19] Tan HY, Ng TW, Liew OW (2010). Accommodating brightness and exposure levels in densitometry of stained polyacrylamide electrophoresis gels. Appl Opt.

[20] Liu R, Han C, Wu D, Xia X, Gu J, Guan H (2015). Prevalence of hyperuricemia and gout in Mainland China from 2000 to 2014: a systematic review and meta-analysis. Biomed Res Int.

[21] Chang FW, Fan HC, Liu JM, Fan TP, Jing J, Yang CL (2017). Estrogen enhances the expression of the multidrug transporter gene ABCG2-increasing drug resistance of breast cancer cells through estrogen receptors. Int J Mol Sci.

[22] Imai Y, Ishikawa E, Asada S, Sugimoto Y (2005). Estrogen-mediated post transcriptional down-regulation of breast cancer resistance protein/ABCG2. Cancer Res.

[23] Allikmets R, Schriml LM, Hutchinson A, Romano-Spica V, Dean M (1998). A human placenta-specific ATP-binding cassette gene (ABCP) on chromosome 4q22 that is involved in multidrug resistance. Cancer Res.

[24] Merino G, van Herwaarden AE, Wagenaar E, Jonker JW, Schinkel AH (2005). Sex-dependent expression and activity of the ATP-binding cassette transporter breast cancer resistance protein (BCRP/ABCG2) in liver. Mol Pharmacol.

[25] Tanaka Y, Slitt AL, Leazer TM, Maher JM, Klaassen CD (2005). Tissue distribution and hormonal regulation of the breast cancer resistance protein (Bcrp/Abcg2) in rats and mice. Biochem Biophys Res Commun.

[26] Hak AE, Choi HK (2008). Menopause, postmenopausal hormone use and serum uric acid levels in US women—the Third National Health and Nutrition Examination Survey. Arthritis Res Ther.

[27] Wang J, Zhu XX, Liu L, Xue Y, Yang X, Zou HJ (2016). SIRT1 prevents hyperuricemia via the PGC-1alpha/PPARgamma-ABCG2 pathway. Endocrine.

[28] Bleau AM, Hambardzumyan D, Ozawa T, Fomchenko EI, Huse JT, Brennan CW (2009). PTEN/PI3K/Akt pathway regulates the side population phenotype and ABCG2 activity in glioma tumor stem-like cells. Cell Stem Cell.

[29] Campbell RA, Bhat-Nakshatri P, Patel NM, Constantinidou D, Ali S, Nakshatri H (2001). Phosphatidylinositol 3-kinase/AKT-mediated activation of estrogen receptor alpha: a new model for anti-estrogen resistance. J Biol Chem.

