# Supplementary material for: Estradiol regulates intestinal ABCG2 to promote urate excretion via the PI3K/Akt pathway
Source: Nutr Metab (Lond). 2021 Jun 18;18:63. doi: 10.1186/s12986-021-00583-y (PMC8212495; doi:10.1186/s12986-021-00583-y)
Supplement: Supplementary file 1 — Additional file 1: Fig. 1. Different time points of EB treated Caco-2 cells. 10−4, 10−6 and 10−8 mol/L estradiol and control(DMSO) groups were set at different time points 24h (A), 48(B) and 72h(C). ABCG2 mRNA expression was significantly induced by 10−4 mol/L EB at 48hrs. Fig. 2. Expression of estrogen receptors A and B (ERA and ERB) on Caco-2 cells. Caco-2 cells naturally express ER, and EB could up-regulate the expression of ERA and ERB in Caco-2 cells. [file 12986_2021_583_MOESM1_ESM.docx]

A B C


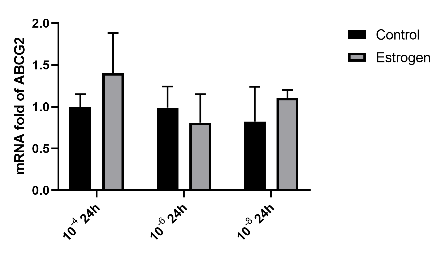




Figure 1 Different time points of EB treated Caco-2 cells. 10^-4^, 10^-6^ and 10^-8^ mol/L estradiol and control(DMSO) groups were set at different time points 24h (A), 48(B) and 72h(C). ABCG2 mRNA expression was significantly induced by 10^-4^ mol/L EB at 48hrs.


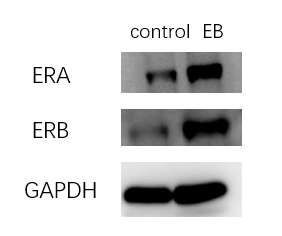


Figure 2 Expression of estrogen receptors A and B (ERA and ERB) on Caco-2 cells. Caco-2 cells naturally express ER, and EB could up-regulate the expression of ERA and ERB in Caco-2 cells.
